# Chemical Constituent Profiling of *Phyllostachys heterocycla* var. Pubescens with Selective Cytotoxic Polar Fraction through EGFR Inhibition in HepG2 Cells

**DOI:** 10.3390/molecules26040940

**Published:** 2021-02-10

**Authors:** Reda F. A. Abdelhameed, Eman S. Habib, Ahmed K. Ibrahim, Koji Yamada, Maged S. Abdel-Kader, Safwat A. Ahmed, Amany K. Ibrahim, Jihan M. Badr, Mohamed S. Nafie

**Affiliations:** 1 Department of Pharmacognosy, Faculty of Pharmacy, Suez Canal University, Ismailia 41522, Egypt; omarreda_70@yahoo.com (R.F.A.A.); emansnd@yahoo.com (E.S.H.); ahmedkhider1993@gmail.com (A.K.I.); safwat_ahmed@pharm.suez.edu.eg (S.A.A.); amany_mohamed@pharm.suez.edu.eg (A.K.I.); jihanbadr2010@hotmail.com (J.M.B.); 2 Garden for Medicinal Plants, Graduate School of Biomedical Sciences, Nagasaki University, Bunkyo-machi 1-14, Nagasaki 852–8521, Japan; kyamada@nagasaki-u.ac.jp; 3 Department of Pharmacognosy, College of Pharmacy, Prince Sattam Bin Abdulaziz University 173, Al-Kharj 11942, Saudi Arabia; 4 Department of Chemistry, Faculty of Science, Suez Canal University, Ismailia 41522, Egypt; mohamed_nafie@science.suez.edu.eg

**Keywords:** apoptosis, *Phyllostachys heterocycla*, polar fraction, cytotoxic activity, EGFR activity

## Abstract

Different extracts of the Bamboo shoot skin *Phyllostachys heterocycla* var. pubescens were screened against panel of cancer cell lines and normal one. The cell viability results exhibited that the ethyl acetate extract showed the least vitality percentage of 2.14% of HepG2 cells. Accordingly, it was subjected to chromatographic separation, which resulted in the isolation of a new natural product; 7-hydroxy, 5-methoxy, methyl cinnamate (**1**), together with four known compounds. The structures of the pure isolated compounds were deduced based on different spectroscopic data. The new compound (**1**) was screened against the HepG2 and MCF-7 cells and showed IC_50_ values of 7.43 and 10.65 µM, respectively. It induced apoptotic cell death in HepG2 with total apoptotic cell death of 58.6% (12.44-fold) compared to 4.71% in control by arresting cell cycle progression at the G1 phase. Finally, compound **1** was validated as EGFR tyrosine kinase inhibitor in both enzymatic levels (IC_50_ = 98.65 nM compared to Erlotinib (IC_50_ = 78.65 nM). Finally, in silico studies of compound **1** through the molecular docking indicated its high binding affinity towards EGFR protein and the ADME pharmacokinetics indicated it as a drug-like.

## 1. Introduction

Cancer is considered as a significant cause of the deaths around the world [1,2]. Treatment of cancer using plant-derived products is an emerging optimistic option that can defeat this aggressive killer. Today, many examples of phytochemicals and their derived analogs are identified as potential candidates for anticancer therapy. Among these examples are vinca alkaloids, epipodophyllotoxins, taxanes, and camptothecin derivatives [3]. Bamboo is a plant of wide occurrence around the world. It includes about 75 genera and 1250 species [4]. The young shoots of bamboo are estimated as one of the most useful health foods as they accumulate a tremendous number of vitamins, proteins, minerals, carbohydrates, and fibers [5,6]. In addition, many therapeutic uses of bamboo leaves were reported as treatment of different cardiovascular diseases and cancer [7]. Previous studies proved the potential of vigorous bamboo leaf extract as a tumor suppressive and cancer-preventive food supplement [8]. In addition, another species, *Phyllostachys edulis,* extract proved to induce apoptosis signaling in osteosarcoma cells, associated with AMPK activation [9]. The present study deals with *Phyllostachys heterocycla* var. pubescens, which is a plant considered as a useful food and its consumption is very common in Southeast Asian and East Asian countries. The methanol extract of the plant was fractionated, and the different extracts were examined for the cytotoxic activity. The ethyl acetate extract revealed the highest cytotoxic activity; accordingly, it was investigated for the main chemical constituents. The chemical structures of the isolated compounds were proved where one of the compounds was found to be new natural product, isolated for the first time from a natural source.

## 2. Results and Discussion

### 2.1. Structure Elucidation of the Isolated Compounds

**Figure 1:**
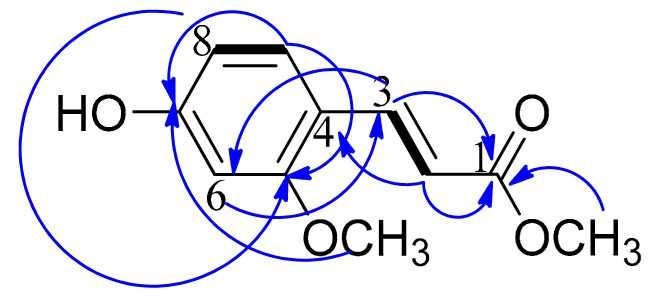
Selected COSY and HMBC correlations of compound **1.**

Compound **1** was isolated as white amorphous powder. Combined spectral data including HRMS, HSQC, ^1^H NMR, and ^13^C NMR established its molecular formula as C_11_H_12_O_4_. Both ^1^H NMR and ^13^C NMR spectra confirmed the presence of a trisubstituted benzene ring. ^1^H NMR revealed three aromatic protons with ABX system resonating at δ_H_ 7.20 (d, *J* = 2.5), 6.70 (dd, *J* = 8.5, 2.5), and 7.80 (d, *J* = 8.5). Two of the aromatic carbons resonated at δ_C_ 160.9 and 168.0, thus confirmed to be oxygenated. The third substituted aromatic carbon was revealed to be substituted with a propenoate moiety. This was clear from the two sp^2^ carbons detected at δ_C_ 114.1 and 145.6 with their corresponding protons resonating at δ_H_ 6.16 and 7.52, respectively. The configuration at the double bond between C-2 and C-3 was determined to be E based on the large coupling constant between H-2 and H-3 (*J* = 15) [10], whereas in the vicinal coupling for Z geometry olefin, *J* = 11 Hz [11]. The ^13^C NMR spectrum revealed the presence of a carbonyl functionality resonating at δ_C_ 169.1 and assigned for C-1. The remaining two carbon signals were proved as methoxyl group from the signals detected at δ_C_ 51.9 and 52.1 with their corresponding protons resonating at δ_H_ 3.70 and 3.78, respectively. A final confirmation of the structure was illustrated by HMBC correlations (Figure 1 and Table 1). Accordingly, compound **1** was assigned as 7-hydroxy, 5-methoxy, methyl cinnamate. According to the available literature, compound **1** is a new natural product reported here for the first time from a natural source. 

**Figure 2:**
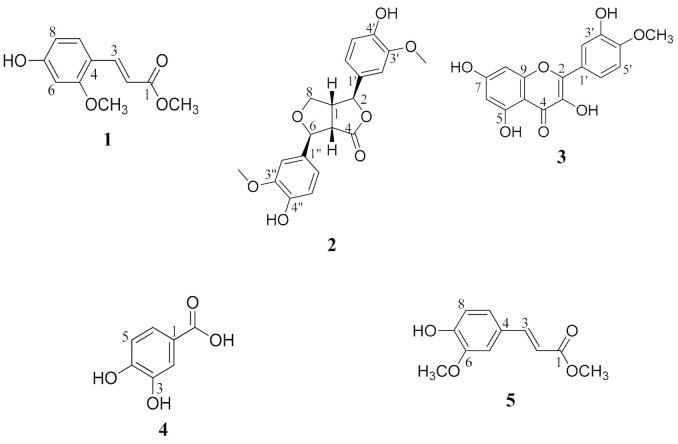
Chemical structures of the isolated compounds.

The chemical structures of compounds **2**–**5** were determined based on different spectroscopic data including 1D and 2D NMR, MS data, as well as comparison of the data with the previously reported in the literature. Compound **2** was elucidated as 4-keto-pinoresinol [12], compound **3** was identified as tamarixetin [13], compound **4** was found to be 3,4-dihydroxybenzoic acid [14], and finally, **5** was methyl ferulate [15,16]. The structures of all the isolated compounds (**1**–**5**) are declared in Figure 2.

### 2.2. Biological Evaluation of the Crude Extract and the Isolated Compounds

#### 2.2.1. Cytotoxic Assay

Crude methanol extract of bamboo shoot skin as well as hexane, ethyl acetate, and butanol extracts were screened against a panel of cancer cell lines HepG2, Hela, A549, and MCF-7 and noncancerous liver cells (THP-1) cells. Percentages of cell viabilities are recorded in Table 2. Crude methanol extract exhibited the least vitality percentage of 6.6% of Hela cells among the tested cell lines at the highest concentrations (100 µg/mL). On the other hand, ethyl acetate extract exhibited the least vitality percentage of 2.14% of HepG2 cells. It is worthy to mention that the 4 known compounds that were isolated from ethyl acetate extract were previously tested for the cytotoxic activity. According to the literature, 4-keto-pinoresinol was reported to be active against SK-Mel-2, B16F1, ovarian cancer SKOV-3, CaoV-3, and cervical cancer HeLa cell lines cycle [17]. Tamarixetin was previously reported to possess cytotoxic action against A-549 and HCC-44 lung adenocarcinoma cells, gastric carcinoma (AGS), skin melanoma (B16F10), brain carcinoma (C6), epitheliod cervix carcinoma (HeLa) cells, MCF-7 and HCT-116 cells [18,19,20]. Additionally, 3,4-dihydrozybenzoic acid was also reported to cause apoptosis on human breast cancer MCF-7 cell, lung cancer A-549 cell, HepG2 cell, cervix HeLa cell, prostate cancer LNCaP cell, and A549 human lung cancer [21,22,23]. Moreover, methyl ferulate previously showed antiproliferative effect on colorectal cancer cells, MCF-7, PC-3, HL-60, MCF-7, and A-549 cells [24,25,26]. This can justify the potent cytotoxic effect of the ethyl acetate extract. In order to complete the profile of ethyl acetate extract activity, the new compound **1** isolated from this extract was screened against the HepG2 and MCF-7 cells and showed IC_50_ values of 7.43 and 10.65 µM, respectively. So, compound **1** was further tested for its apoptotic activity against HepG2 cells. 

#### 2.2.2. Annexin V/PI Staining

**Figure 3:**
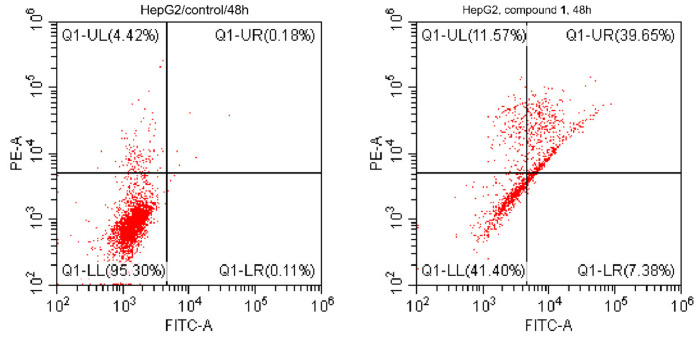
Cytograms annexin-V/propidium iodide stained HepG2 cells showing induction of apoptosis in cancer cells by compound 1 (IC50 = 7.43 µM, 48 h) compared to untreated cells. “Quadrant charts show Q-UL (intermediate, AV–/PI+), Q-UR (late apoptotic cells, AV+/PI+), Q-LL (normal cells, AV–/PI–), and Q-LR (early apoptotic cells, AV+/PI–).”

Staining with Annexin V/PI on HepG2 cells treated with compound **1** (IC_50_ = 7.43 µM, 48 h) and untreated cells for investigating the apoptotic activity of its treatment in the HepG2 cells was done. As shown in Figure 3, results indicated that the treatment caused apoptosis in HepG2 cells with total apoptotic cell death of 58.6% (12.44-fold) compared to 4.71% in control. It induced apoptosis by 7.38%, (early), 11.57% (intermediate), and 39.65% (late). These results are with good evidence for apoptosis induction in the HepG2-treated cells.

#### 2.2.3. Cell Cycle Analysis

**Figure 4:**
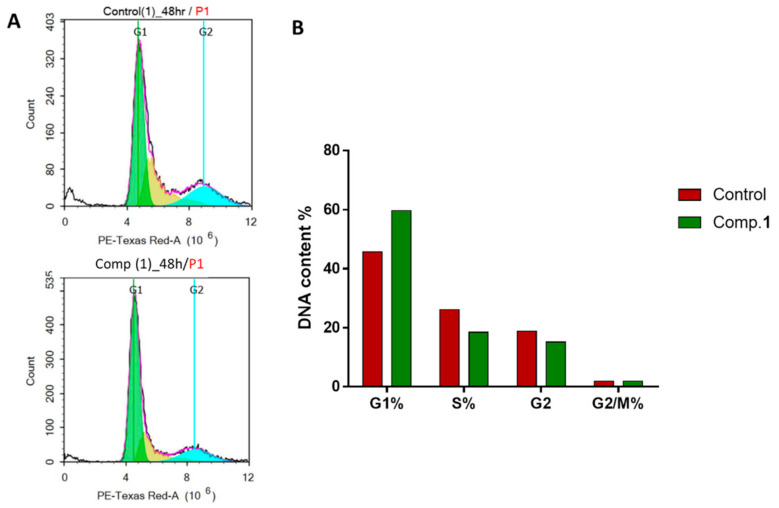
(**A**) Cytogram of the cell cycle distribution of untreated and treated HepG2 cells with. compound **1** (7.43 µM, 48 h); (**B**) bar chart representation of the percentage of cell population in different HepG2 cell cycle phases.

A significant test that shows the proportion of cell proliferation in each phase of cell development following treatment with cytotoxic compound is the cell cycle analysis. Therefore, to analyze the cell cycle kinetics of the HepG2 cells, the cells were treated with compound **1** (IC_50_ = 7.43 µM, 48 h). As shown in Figure 4, compound **1** treatment showed an increase at G1-phase cell-cycle arrest with 1.3-fold (59.63%, compared to 45.75% for control), while it decreased the cell population distribution at S (18.46 compared to 26.20%), G2 (15.22 compared to 18.77%), and G2/M (1.88 compared to 1.89%) phases; this may have resulted in compound **1** treatment-induced cytotoxic activity in HepG2 cells by arresting cell cycle progression at the G1 phase. These results agreed with our previous studies [27,28], which investigated the apoptotic activity of some solvent extracts using the flow cytometric analyses.

### 2.3. In Silico Studies

#### 2.3.1. EGFR Inhibition Activity

**Figure 5:**
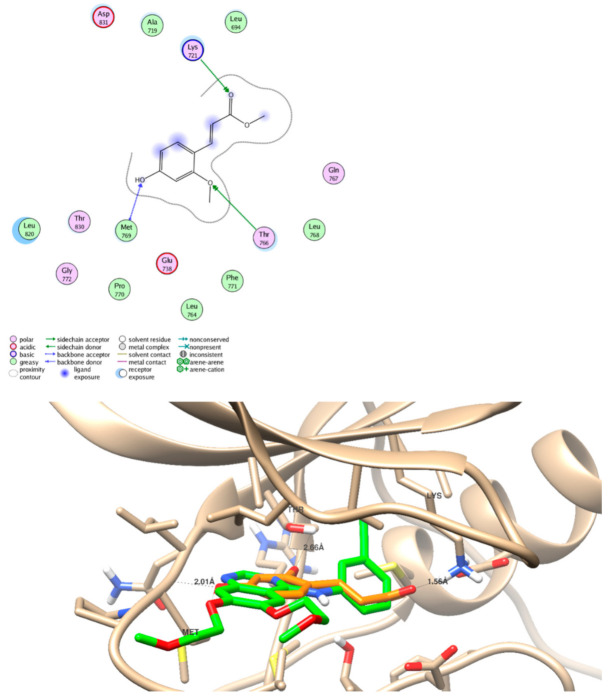
Representative of superimposition and binding mode of compound **1** (Orange) and the cocrystalized ligand (Green) inside the epidermal growth factor receptor tyrosine kinase (EGFR)-binding site (PDB 1M17) in 2D and 3D.

The five identified compounds were screened for their binding activities through molecular docking studies, they were docked inside the EGFR binding site (PDB = 1M17) with binding energies (−9.74 to −18.82 Kcal/mol) and formed strong hydrogen bond interactions with the key amino acid Met 769. All interactions between moieties of ligand and receptor are summarized in Table 3 and Figure 5. The new compound **1** formed one hydrogen bond through its carboxylic group as HBA with binding energy of −18.82 Kcal/mol. Interestingly, compound **3** forms three hydrogen bonds with Met 769 as HBD and HBA through the hydroxylic and carbonyl groups, respectively. Since the molecular docking results exhibited good binding activity towards the EGFR protein, the new compound **1** was further tested against the EGFR enzymatic assay; it exhibited good inhibitory activity with IC_50_ value of 98.65 nM compared to erlotinib (IC_50_ = 78.65 nM). The agreement of the in silico and in situ results validate the EGFR inhibitor activity.

#### 2.3.2. ADME Pharmacokinetics

**Figure 6:**
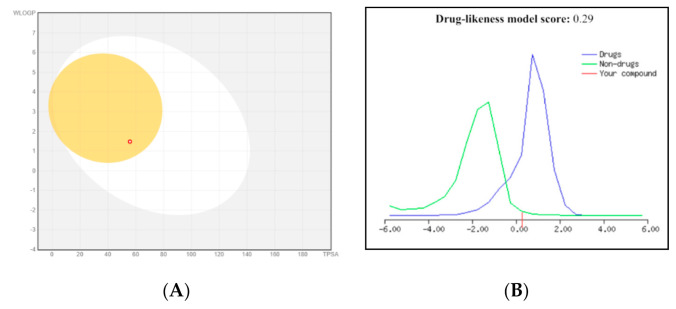
(**A**) BOILED-Egg model for compound **7** using SwissADME: “points located in the BOILED-Egg‘s yolk are molecules predicted to passively permeate through the blood–brain barrier (BBB), while points located in the BOILED-Egg‘s white are molecules predicted to be passively absorbed by gastrointestinal (GI) tract” and (**B**) drug likeness score of compound **1** using MolSoft: “The green color means nondrug-like behavior, and those fall under blue color area are considered as drug-like. Those compounds having negative or zero value should not be considered as drug like.”

The five identified compounds were screened for their ADME pharmacokinetics as previously described [29] (Table 4 and Figure 6). All tested compounds possess accepted values of H-bond donors (1–4) and H-bond acceptors (3–7), which are following the right criteria for hydrogen-bonding capacity for good drug permeability [30]. When the H-bond donors exceed 5, and the H-bond acceptors reach 10, drugs will be poorly absorbed. All compounds have log *p* values ≤ 5, so they had good membrane permeability. All of the compounds obeyed to Lipinski’s five rule, so, they could, therefore, be considered as drugs applicants for oral absorption. Furthermore, compounds **1**, **3,** and **4** showed positive values for drug-likeness scores, which indicated them as drug-like.

## 3. Experimental

### 3.1. General Experimental Procedures

1D and 2D NMR spectra (chemical shifts in ppm, coupling constants in Hz) were recorded on Bruker Avance DRX 500 MHz spectrometers (MA, USA). HRMS were determined by direct injection using Thermo Scientific UPLC RS Ultimate 3000-Q Exactive (Thermo Fisher Scientific, Waltham, MA, USA) hybrid quadrupole-Orbitrap mass spectrometer combined with high-performance quadrupole precursor selection with high-resolution, accurate-mass (HR/AM) Orbitrap™ detection. Detection was done in both positive and negative modes separately. Column chromatographic separations were carried out using Sephadex LH-20 (0.25–0.1 mm, Pharmacia, Sigma-Aldrich, St. Louis, Missouri, USA) and silica gel 60 (0.04–0.063 mm). TLC was accomplished using TLC plates precoated with silica gel 60 F_254_ (0.2 mm, Merck, NY, USA). Spots were visualized by UV absorption at λ of 255 and 366 nm followed by spraying with *P*-anisaldehyde/H_2_SO_4_. 

### 3.2. Plant Material

*Phyllostachys heterocycla* var. pubescens was harvested in Isahaya, Nagasaki, Japan and collected on October 2011. The plant was stored at −24 °C until used. It was identified by Koji Yamada, Garden for Medicinal Plants, School of Pharmacy, Nagasaki University, Japan. A voucher specimen was kept under registration number KY-11 in the herbarium of Pharmacognosy Department, Faculty of Pharmacy, Suez Canal University, Ismailia, Egypt. 

### 3.3. Extraction and Purification of Compounds ***1**–**5***

An amount of 12.7 kg was repeatedly extracted with methanol (20 L) followed by further extraction with CHCl_3_: MeOH (1:1) (20 L) at room temperature and the combined extracts were concentrated in vacuo to give a residue of 110 g. The residue was suspended in H_2_O (4 L) and extracted with *n*-hexane, EtOAc, *n*-BuOH, successively. The EtOAc extract was subjected to SiO_2_ column, eluted with CHCl_3_/MeOH gradient, and monitored by TLC to compile the resulted similar fractions. Based on TLC analysis, two fractions were subjected to further investigation.

First fraction was applied over SiO_2_ column with gradual elution using MeOH: CHCl_3_. Subfractions eluted were investigated and similar ones were combined to afford three main subfractions. Subfraction 1 was chromatographed on silica gel column packed in CHCl_3_ and eluted with a step gradient of MeOH: CHCl_3_ to afford semipure compound that was finally purified over Sephadex LH-20 column and eluted with CHCl_3_: MeOH (1:1) to obtain pure compound **2** (1.8 mg). Subfraction 2 was purified by dissolving it in CHCl_3_: MeOH (1:1) followed by decantation and crystallization to obtain the pure compound **3** (2.3 mg). Subfraction 3 was purified on preparative TLC using CHCl_3_: MeOH (20:1) and a drop of glacial acetic acid to yield pure compound **4** (3.3 mg). The second fraction was chromatographed over Sephadex LH-20 column and eluted with CHCl_3_: MeOH (1:1) followed by purification on preparative TLC using CHCl_3_: MeOH (20:1) as developing solvent mixture to obtain two pure compounds; compound **5** (11 mg) and compound **1** (23.6 mg). 

### 3.4. Spectroscopic Data of the Isolated Compounds

7-Hydroxy, 5-methoxy, methyl cinnamate (**1**): White amorphous powder; HRMS analysis (negative mode) *m*/*z*: 207.0658 [M − H]^−^, molecular formula: C_11_H_12_O_4_, NMR data: see Table 1. 

4-Keto-pinoresinol (**2**): White amorphous powder; ^1^H-NMR (500 MHz, CD_3_OD): δ_H_ = 6.79: 6.98 (6H, m, aromatic-H), 5.79 (1H, s, -OH), 5.78 (1H, s, -OH), 3.34 (1H, m, H-1), 5.24 (1H, d, *J* = 5, H-2), 3.68 (1H, dd, *J* = 5, 10, H-5), 5.40 (1H, d, *J* = 5, H-6), 4.29 (1H, dd, *J* = 5, 10, H-8a), 4.03 (1H, dd, *J* = 5, 10 H-8b), 3.88 (3H, s, OCH_3_), 3.87 (3H, s, OCH_3_). ^13^C-NMR (125 MHz, CD_3_OD): δ_C_ = 51.0 (C-1), 85.1 (C-2), 179.9 (C-4), 54.5 (C-5), 87.2 (C-6), 73.8 (C-8), 132.4 (C-1′), 110.6 (C-2′), 149.2 (C-3′), 147.5 (C-4′), 116.2 (C-5′), 119.5 (C-6′), 133.2 (C-1′′), 110.7 (C-2′′), 149.4 (C-3′′), 148.2 (C-4′′), 116.4 (C-5′′), 119.8 (C-6′′), 56.4 (3′-*OCH_3_*), 56.5 (3′′-OCH_3_). 

Tamarixetin (**3**): Yellow powder; ^1^H-NMR (500 MHz, DMSO-*d*_6_): δ_H_ 6.29, brs (H-6), 6.64, brs (H -8), 7.39, brs, (H-2′), 7.06, brs, (H-5′), 7.41, brs, (H-6′), 3.88, s, (OCH_3_), 13.05 (OH). ^13^C-NMR (125 MHz, DMSO-*d*_6_): δ_C_ 148.9 (C-2), 136.0 (C-3), 177.0 (C-4), 162.2 (C-5), 99.5 (C-6), 165.1 (C-7), 94.8 (C-8), 153.9 (C-9), 104.8 (C-10), 123.0 (C-1′), 114.1 (C-2′), 146.3 (C-3′), 149.0 (C-4′), 119.5 (C-5′), 123.8 (C-6′), 56.9 (OCH_3_). 

3,4-Dihydroxybenzoic acid (**4**): White amorphous powder; ^1^H-NMR (500 MHz, CD_3_OD): δ_H_ 7.44, brs (H-2), 6.81, d, *J* = 10.0 (H-5), 7.46, brd, *J* = 10.0 (H-6). ^13^C-NMR (125 MHz, CD_3_OD): δ_C_ 122.0 (C-1), 116.4 (C-2), 144.6 (C-3), 150.0 (C-4), 114.5 (C-5), 122.5 (C-6), 169.9 (CO).

Methyl ferulate (**5**): White amorphous powder; ^1^H-NMR (500 MHz, CDCI_3_): δ_H_ 6.18, d, *J* = 15.9 (H-2), 7.41, d, *J* = 15.9 (H-3), 6.80, brs (H-5), 6.89, d, *J* = 8.4 (H-8), 7.52, brd, *J* = 8.4 (H-9), 4.10, s (1-OCH_3_), 4.20, s, (6-OCH_3_). ^13^C-NMR (125 MHz, CDCI_3_): δ_C_ 167.9 (C-1), 114.8 (C-2), 145.0 (C-3), 126.8 (C-4), 109.5 (C-5), 148.1 (C-6), 146.9 (C-7), 114.8 (C-8), 123.1 (C-9), 51.9 (1-OCH_3_), 55.8 (6-OCH_3_). 

### 3.5. Biological Evaluation of the Compounds

#### 3.5.1. Cytotoxic Activity

Cell viability using the MTT assay was performed to investigate the effect of the crude extract, different extracts of hexane, ethyl acetate, and butanol and pure of compound **1** [31]. Full methodology is supported in the Supplementary File.

#### 3.5.2. Apoptotic Investigation Using Flow Cytometric Analysis

Cell cycle analysis and apoptotic assays were performed to determine in which phase cells would be arrested and to calculate the percentage of apoptotic activity. Full methodologies of flow cytometric assays are provided in the Supplementary File [32]. After treatment with compound **1** (IC_50_ = 7.43 μM, 48 h), the harvested HepG2 cells were subjected to flow cytometric analyses including “FITC/Annexin-V-FITC/PI differential apoptosis/necrosis assessment and DNA content flow cytometry aided cell cycle analysis.”

#### 3.5.3. EGFR Inhibition Activity

EGFR-TK assay was performed to evaluate the inhibitory potency of novel compound **1** against the EGFR [33]. For full methodology see the Supplementary File.

#### 3.5.4. In Silico Studies

##### Molecular Docking Simulation

For the docking studies, the crystal structure of the epidermal growth factor receptor tyrosine kinase (EGFR) was obtained from the Protein data bank (PDB code: 1M17). According to routine work [34], preparation and optimization of ligand and receptor were carried out. All molecular modeling studies were conducted on a computational software basis using the “Molecular Operating Environment (MOE 2014-09 Chemical Computing Group, Canada)”. Each ligand–receptor complex was tested for interaction analysis, 2D images were made using the MOE visualizing tool, and 3D images were taken by Chimera as a visualizing software [35].

##### ADME Pharmacokinetics 

ADME pharmacokinetics parameters of the most active compounds were calculated using a set of software including “MolSoft,” “Molinspiration”, and “SwissADME” websites as previously described by Youssef et al. 2020 [36].

### 3.6. Flow Cytometric Analysis

#### 3.6.1. FITC/Annexin-V-FITC/PI Differential Apoptosis/Necrosis Assessment

Apoptosis and necrosis cell populations are determined using Annexin V-FITC apoptosis detection kit (Abcam Inc., Cambridge Science Park, Cambridge, UK) coupled with 2 fluorescent channels flowcytometry. After treatment with test compounds for 48 h, cells (10^5^ cells) are collected by trypsinization and washed twice with ice-cold PBS (pH 7.4). Then, cells are incubated in dark with 0.5 mL of Annexin V-FITC/PI solution for 30 min in dark at room temperature according to manufacturer protocol. After staining, cells are injected via ACEA Novocyte™ flowcytometer (ACEA Biosciences Inc., San Diego, CA, USA) and analyzed for FITC and PI fluorescent signals using FL1 and FL2 signal detector, respectively (λex/em 488/530 nm for FITC and λex/em 535/617 nm for PI). For each sample, 12,000 events are acquired and positive FITC and/or PI cells are quantified by quadrant analysis and calculated using ACEA NovoExpress™ software (ACEA Biosciences Inc.,San Diego, CA, USA).

#### 3.6.2. DNA Content-Flow Cytometry Aided Cell Cycle Analysis

After treatment with test compounds for 48 h, cells (10^5^ cells) are collected by trypsinization and washed twice with ice-cold PBS (pH 7.4). Cells are re-suspended in two milliliters of 60% ice-cold ethanol and incubated at 4 °C for 1 h for fixation. Fixed cells are washed twice again with PBS (pH 7.4) and re-suspended in 1 mL of PBS containing 50 μg/mL RNAase A and 10 μg/mL propidium iodide (PI). After 20 min of incubation in dark at 37 C, cells are analyzed for DNA contents using flow cytometry analysis using FL2 (λex/em 535/617 nm) signal detector (ACEA Novocyte™ flowcytometer, ACEA Biosciences Inc., San Diego, CA, USA). For each sample, 12,000 events are acquired. Cell cycle distribution is calculated using ACEA NovoExpress™ software (ACEA Biosciences Inc., San Diego, CA, USA).

### 3.7. EGFR Inhibitory Assay

EGFR-TK assay was performed to evaluate the inhibitory potency of novel compound **1** against EGFR. Baculoviral expression vectors including pBlueBacHis2B and pFASTBacHTc were used separately to clone 1.6 kb cDNA coding for EGFR cytoplasmic domain (EGFR-CD, amino acids 645–1186). 5ʹ upstream to the EGFR sequence comprised a sequence that encoded (His)_6_. HepG2 cells were infected for 48h for protein expression. The pellets of HepG2 cells were solubilized in a buffer containing sodium vanadate (100 µM), aprotinin (10 µg/mL), triton (1%), HEPES buffer (50 mM), ammonium molybdate (10 µM), benzamidine HCl (16 µg/mL), NaCl (10 mM), leupeptin (10 µg/mL) and pepstatin (10 µg/mL) at 0 °C for 20 min at pH 7.4, followed by centrifugation for 20 min. To eliminate the nonspecifically bound material, a Ni-NTA superflow packed column was used to pass through and wash the crude extract supernatant first with 10 mM and then with 100 mM imidazole. Histidine-linked proteins were first eluted with 250 and then with 500 mM imidazole subsequent to dialysis against NaCl (50 mM), HEPES (20 mM), glycerol (10%) and 1 µg/mL each of aprotinin, leupeptin and pepstatin for 120 min. The purification was performed either at 4 °C or on ice. To record autophosphorylation level, EGFR kinase assay was carried out on the basis of DELFIA/Time-Resolved Fluorometry. The compound was first dissolved in DMSO absolute, subsequent to dilution to appropriate concentration using HEPES (25 mM) at pH 7.4. Each compound (10 µL) was incubated with recombinant enzyme (10 µL, 5 ng for EGFR, 1:80 dilution in 100 mM HEPES) for 10 min at 25 °C, subsequent to the addition of 5X buffer (10 µL, containing 2 mM MnCl_2_, 100 µM Na_3_VO_4_, 20 mM HEPES and 1 mM DTT) and ATP-MgCl_2_ (20 µL, containing 0.1 mM ATP and 50 mM MgCl_2_) and incubation for 1h. The negative and positive controls were included in each plate by the incubation of enzyme either with or without ATP-MgCl_2_. The liquid was removed after incubation and the plates were washed thrice using wash buffer. Europium-tagged antiphosphotyrosine antibody (75 µL, 400 ng) was added to each well followed by incubation of 1h and then washing of the plates using buffer. The enhancement solution was added to each well and the signal was recorded at excitation and emission wavelengths of 340 at 615 nm. The autophosphorylation percentage inhibition by compounds was calculated using the following equation:

100% − [(negative control)/(positive control) – (negative control)](1)

Using the curves of percentage inhibition of five concentrations of each compound, IC_50_ was calculated. Majority of signals detected by antiphosphotyrosine antibody were from EGFR because the enzyme preparation contained low impurities.

## 4. Conclusions

The ethyl acetate extract of *Phyllostachys heterocycla* var. pubescens exhibited potent cytotoxic activity and showed vitality percentage of 2.14% of HepG2 cells. This extract afforded five compounds of which one new compound was identified as 7-hydroxy, 5-methoxy, methyl cinnamate. The new compound was screened against the HepG2 and MCF-7 cells and exhibited IC_50_ values of 7.43 and 10.65 µM, respectively, with non-cytotoxic activity against the THLE2 cells. It induced total apoptotic cell death of 58.6% (12.44-fold) compared to 4.71% in control by arresting cell cycle progression at the G1 phase. Moreover, the EGFR activity was validated by the mechanism of action through both enzymatic and in silico levels. The study demonstrated the edible plant *Phyllostachys heterocycla* var. pubescens as an excellent promising anticancer agent.

## Figures and Tables

**Table 1:** NMR spectroscopic data of compound **1** (CDCl_3_, 500 and 125 MHz).

Position	δ_C_ (m) ^a^	δ_H_ (m, *J* in Hz)
1	169.1 (C)	
2	114.1 (CH)	6.16 (d, *J* = 15)
3	145.6 (CH)	7.52 (d, *J* = 15)
4	126.3 (C)	
5	160.9 (C)	
6	130.1 (CH)	7.20 (d, *J* = 2.5)
7	168.0 (C)	
8	115.4 (CH)	6.70 (dd, *J* = 8.5, 2.5)
9	131.9 (CH)	7.80 (d, *J* = 8.5)
1- OCH_3_	51.9 (CH_3_)	3.70 (s)
5- OCH_3_	52.1 (CH_3_)	3.78 (s)

^a^ Multiplicities were deduced from multiplicity-edited HSQC.

**Table 2:** Percentages of cell viabilities of crude, solvent fractions, and identified compounds against four cancerous cell lines HepG2, Hela, A549, and MCF-7 and noncancerous cells.

Extracts	% of Cell Viability at 100 µg/mL				
	HepG2	Hela	A549	MCF-7	THP-1
Crude methanol extract	47.5 ± 0.76	6.6 ± 0.89	33.18 ± 0.19	36.6 ± 0.64	86 ± 1.67
Hexane extract	58.6 ± 1.26	64.53 ± 1.23	52.63 ± 1.14	49.8 ± 0.81	96 ± 1.98
Ethyl acetate extract	2.14 ± 0.15	24.23 ± 1.52	15.05 ± 0.57	12.14 ± 0.81	86 ± 0.98
Butanol extract	84.3 ± 1.98	94.31 ± 0.09	94.26 ± 0.23	63.7 ± 0.36	87 ± 1.04
	IC_50_ ± SD *^#^				
Compound **1** (µM)	7.43 ± 0.82	ND	ND	10.65 ± 1.01	≥50

* Values are expressed as mean ± SD of 3 independent trials (n = 3). **^#^** IC_50_ were calculated using GraphPad Prism **7** software using nonlinear regression Dose-Inhibition curve fit.

**Table 3:** Ligand–receptor interactions of the docked compounds with the binding energy (Kcal/mol) inside the 1M17 as epidermal growth factor receptor tyrosine kinase (EGFR) inhibitors

Compounds	In Silico Molecular Docking Simulation as EGFR Inhibitors			In Vitro EGFR Activity(nM) *
	Binding Energy (Kcal/mol)	HB Interactions with The Key Amino Acid (Met 769)		
1	−18.82	HBA as	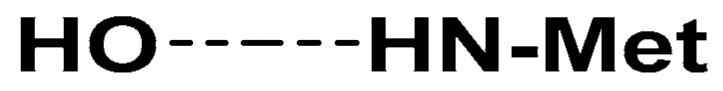	98.65 ± 9.87
2	−16.28	HBA as	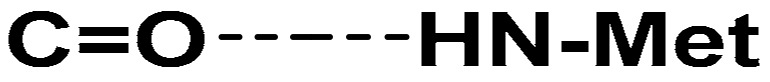	–
3	−13.2	HBD as	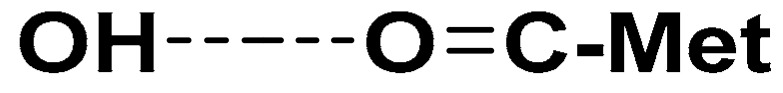	–
		HBA as	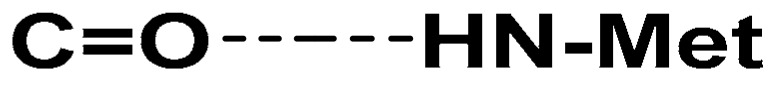	–
4	−9.74	HBA as	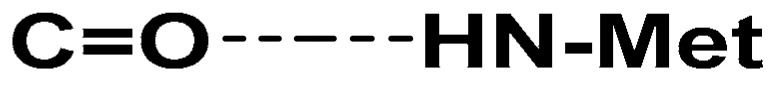	–
5	−16.77	HBA as	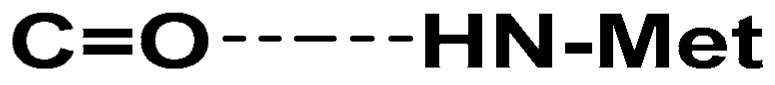	–
Erlotinib	–	–		78.65 ± 6.54

* Values are expressed as mean ± SD of 3 independent triplicates and are calculated by GraphPad Prism 7.

**Table 4:** Molecular properties of and drug-likeness.

Website	Compound ADME	1	2	3	4	5
Molinspiration 2018.10	Mwt (D)	208.21	372.37	316.26	154.12	208.21
	MV (A^3^)	189.55	321.96	257.61	127.08	189.55
	PSA (A^2^)	55.77	94.46	120.36	77.75	55.77
	Log p	1.85	2.46	1.99	0.88	1.86
	nrotb	4	4	2	1	4
	nviolations	0	0	0	0	0
MolSoft	HBA	3	7	7	4	4
	HBD	1	2	4	3	1
	Solubility (mg/L)	375.19	2726.4	1102.6	3378.7	1461.2
	Drug-likeness score	0.29	−0.09	0.16	0.23	−0.76

Mwt: molecular weight, MV: molecular volume, PSA: polar surface area, Log p: Log P: octanol-water partition coefficient, nrotb: number of rotatable bonds, nviolations: number of violations, HBA: hydrogen bond acceptor, HBD: hydrogen bond donor [29,30].

## Data Availability

No new data were created or analyzed in this study. Data sharing is not applicable to this article. The data presented in this study are available in Supplementary Materials.

## Supplementary Materials

Figure S1. 1HNMR spectrum of compound (**1**); Figure S2. 13CNMR spectrum of compound (**1**); Figure S3. COSY spectrum of compound (**1**); Figure S4. HSQC spectrum of compound (**1**); Figure S5. HMBC spectrum of compound (**1**); Scheme 1. Mass spectrum of compound (**1**).
