# Supplementary material for: Chemical Constituent Profiling of Phyllostachys heterocycla var. Pubescens with Selective Cytotoxic Polar Fraction through EGFR Inhibition in HepG2 Cells
Source: Molecules. 2021 Feb 10;26(4):940. doi: 10.3390/molecules26040940 (PMC7916669; doi:10.3390/molecules26040940)
Supplement: Supplementary file 1 [file molecules-26-00940-s001.pdf]

Supplementary materials

Chemical Constituent Profiling of *Phyllostachys heterocycla* var. Pubescens with Selective Cytotoxic Polar Fraction through EGFR Inhibition in HepG2 Cells

Reda F.A. Abdelhameed [1,†], Eman S. Habib [1,†], Ahmed K. Ibrahim [1], Koji Yamada [2], Maged S. Abdel-Kader [3,*], Safwat A. Ahmed [1], Amany K. Ibrahim [1], Jihan M. Badr [1] and Mohamed S. Nafie [4]

[1] Department of Pharmacognosy, Faculty of Pharmacy, Suez Canal University, Ismailia 41522, Egypt; omarreda_70@yahoo.com (R.F.A.A.); emansnd@yahoo.com (E.S.H.); ahmedkhider1993@gmail.com (A.K.I.); safwat_ahmed@pharm.suez.edu.eg (S.A.A.); amany_mohamed@pharm.suez.edu.eg (A.K.I.); jihanbadr2010@hotmail.com (J.M.B.)

[2] Garden for Medicinal Plants, Graduate School of Biomedical Sciences, Nagasaki University, Bunkyo-machi 1-14, Nagasaki 852–8521, Japan; kyamada@nagasaki-u.ac.jp

[3] Department of Pharmacognosy, College of Pharmacy, Prince Sattam Bin Abdulaziz University 173, Al-Kharj 11942, Saudi Arabia

[4] Department of Chemistry, Faculty of Science, Suez Canal University, Ismailia 41522, Egypt; mohamed_nafie@science.suez.edu.eg

* Correspondence: mpharm101@hotmail.com

† These authors have equal contribution to this work.

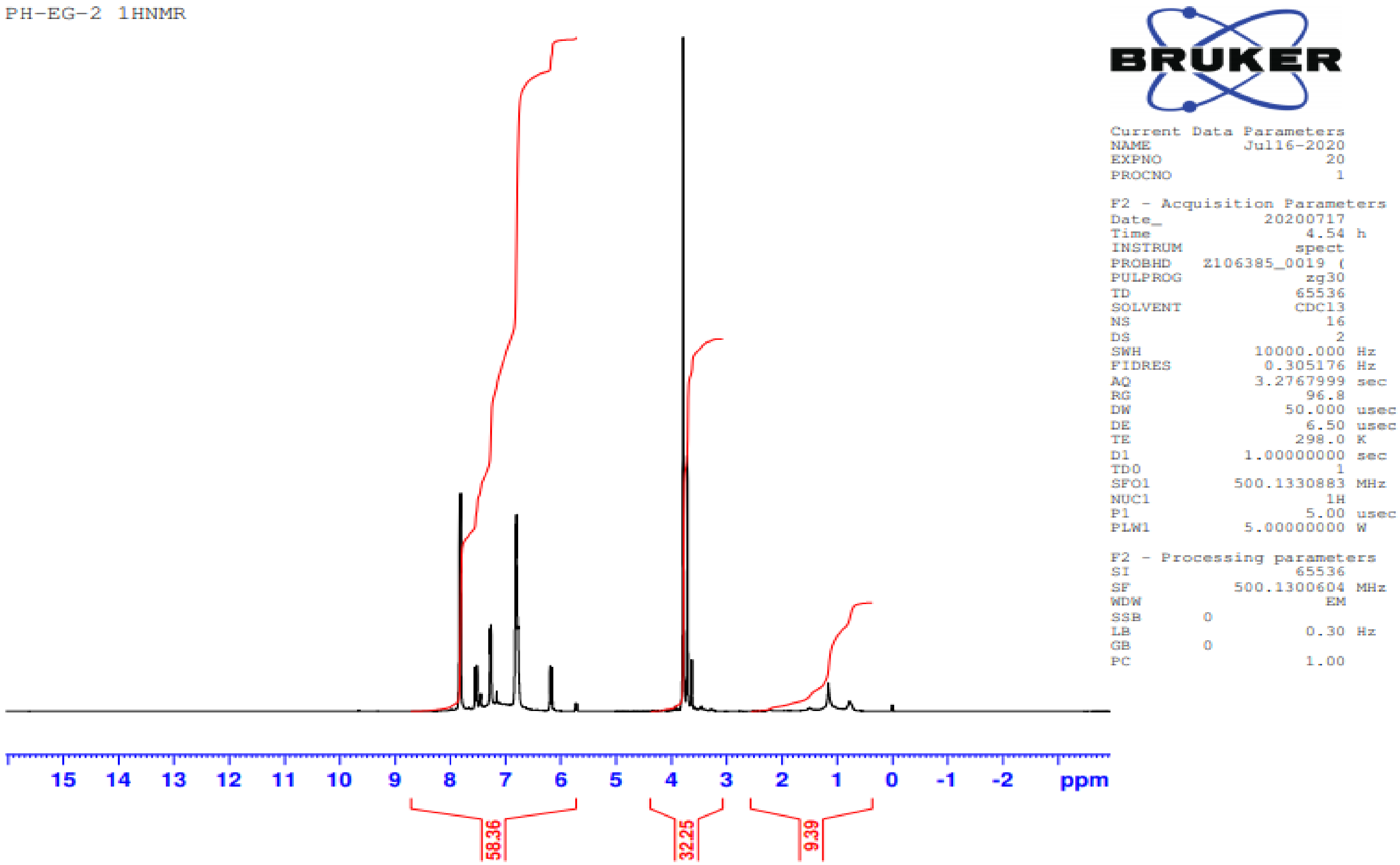

Activ
Go to

Figure S1. [1]**HNMR spectrum of compound (1).**

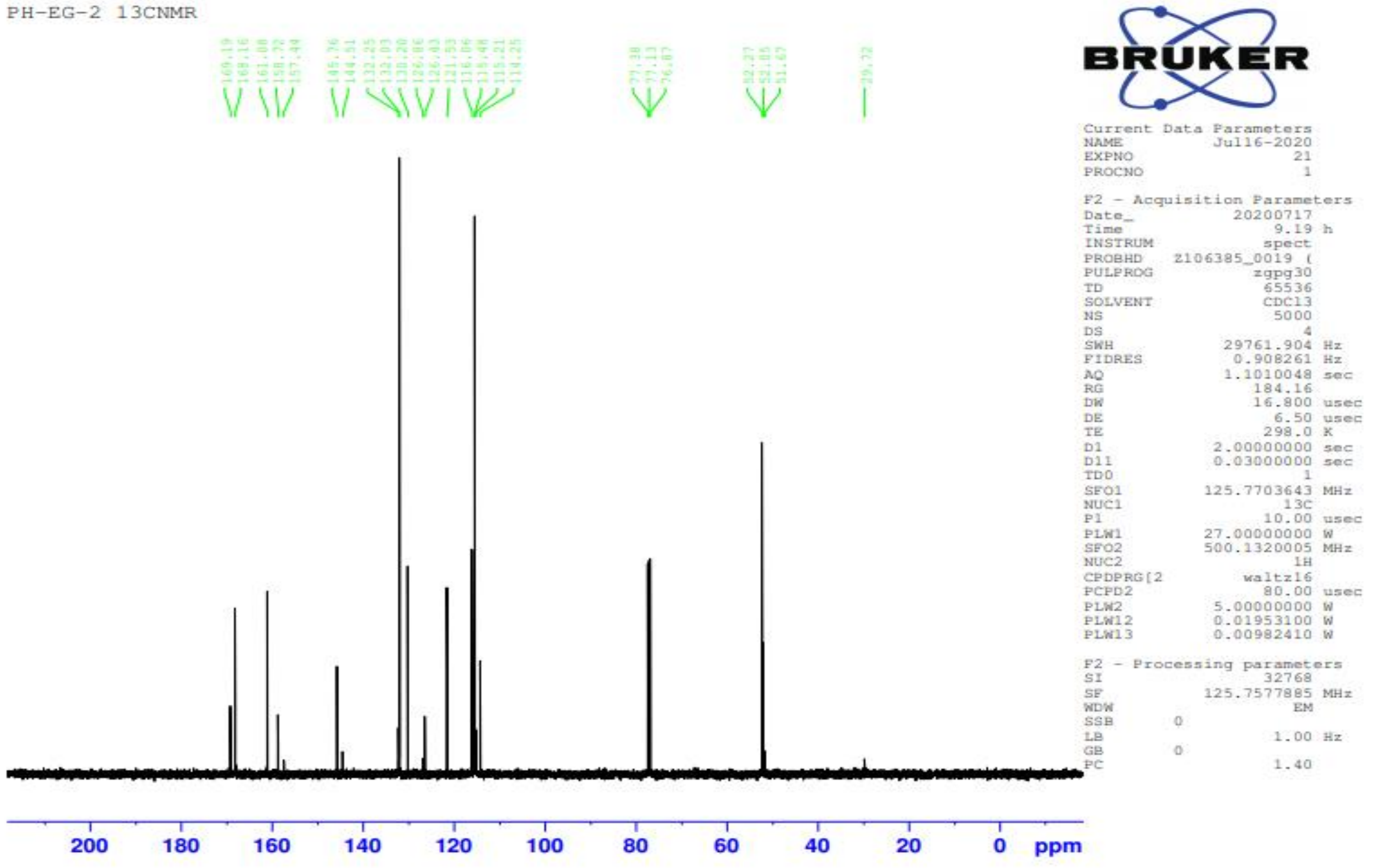

Figure S2. ^{13}CNMR spectrum of compound (1).

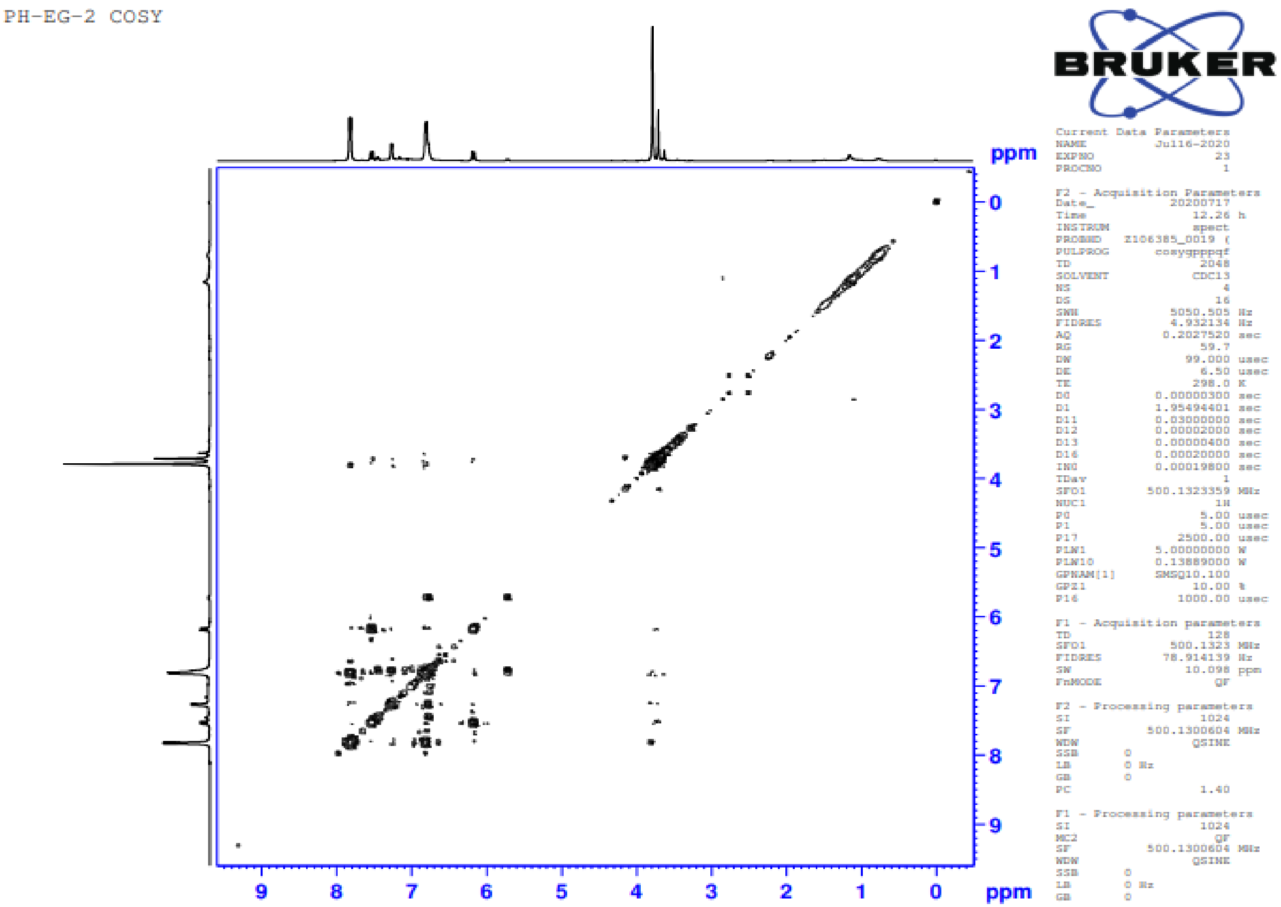

Ac
Go

Figure S3. COSY spectrum of compound (1).

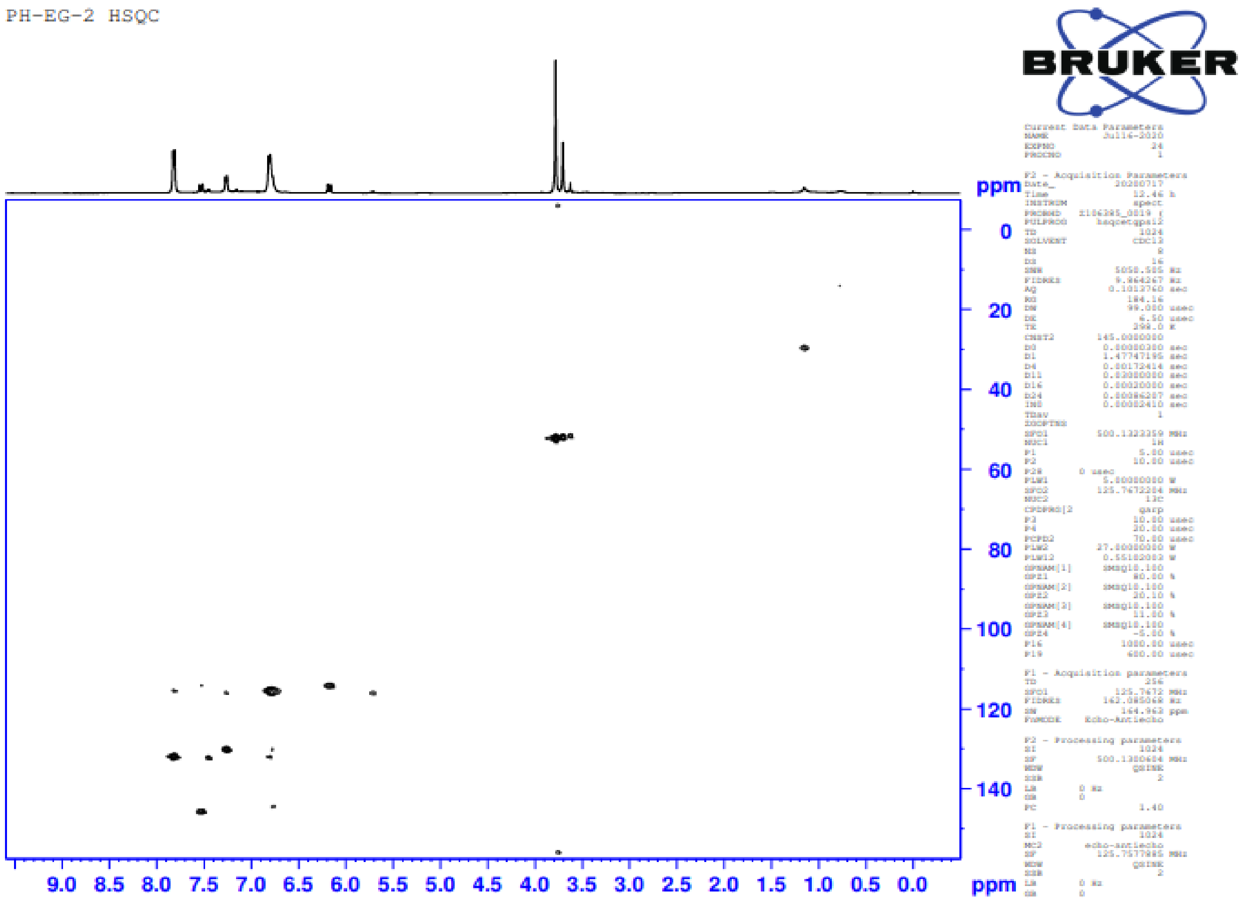

Act
Go

Figure S4. HSQC spectrum of compound (1).

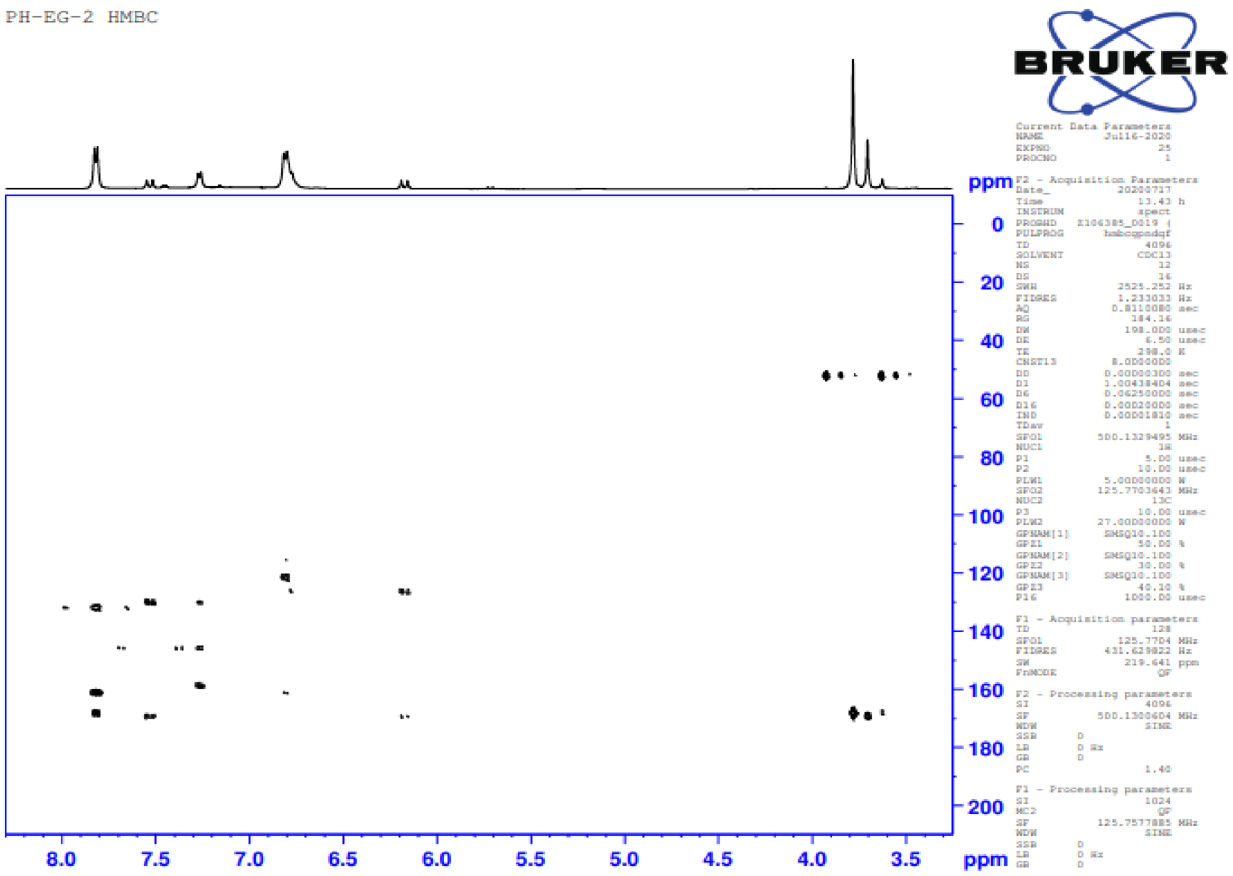

Figure S5. HMBC spectrum of compound (1).

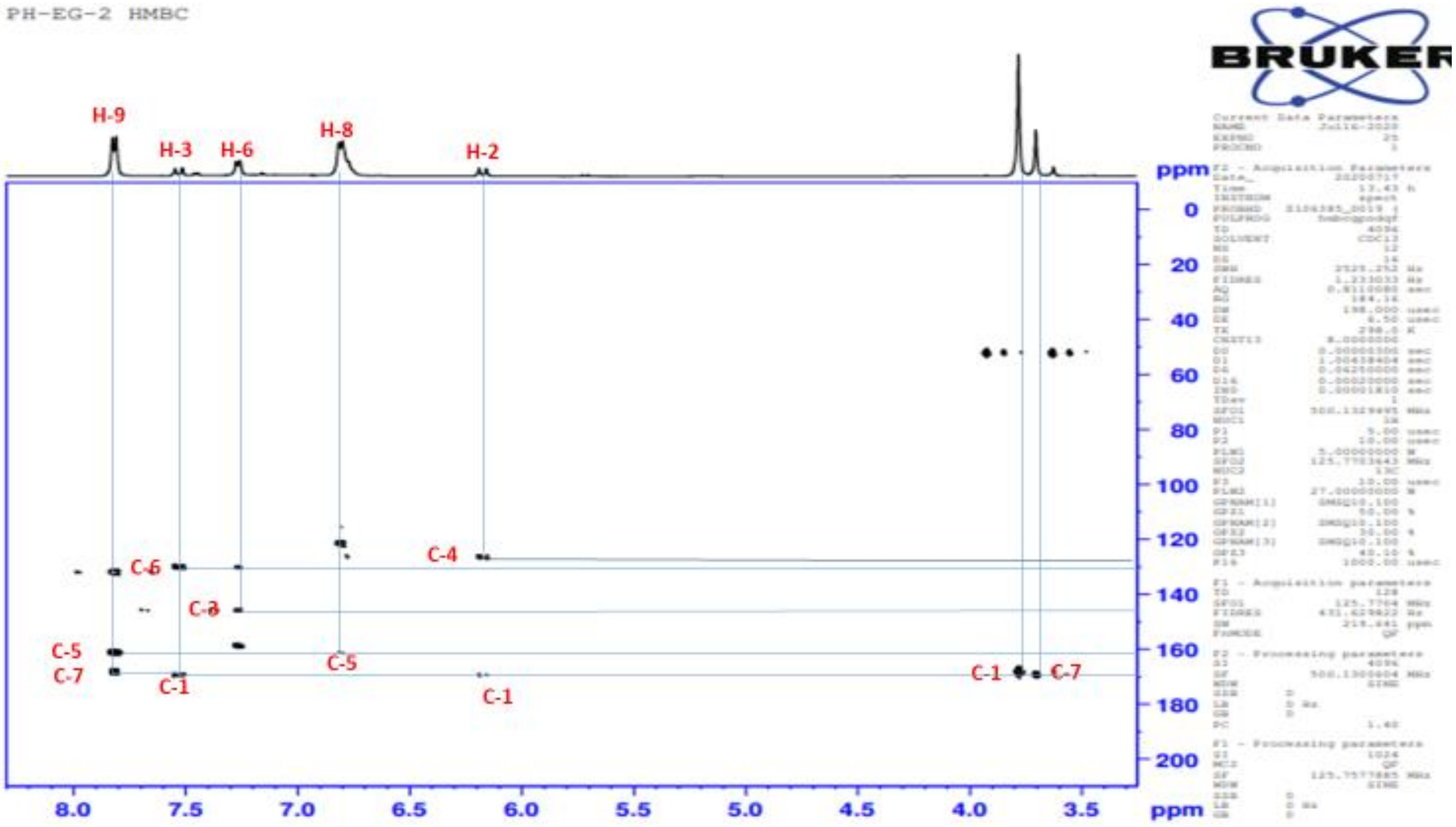

Scheme 1. Mass spectrum of compound (**1**).
